# Case Report: Spontaneous simultaneous coronary and carotid dissection in a young cannabis user

**DOI:** 10.12688/f1000research.52606.2

**Published:** 2021-06-11

**Authors:** Hassen Ibn Hadj Amor, Imen Touil, Seif Boukriba, Skander Bouchnak, Salma Kraiem, Ramzi Rouabhia

**Affiliations:** 1 Cardiology Department, Taher Sfar university hospital, Mahdia, 5100, Tunisia; 2 Pneumology Department, Taher Sfar university hospital, Mahdia, 5100, Tunisia; 3 Radiology Department, Rabta University Hospital, Tunis, 1007, Tunisia

**Keywords:** Cannabis, coronary dissection, carotid dissection, acute coronary syndrome, ischemic stroke.

## Abstract

Due to legalization of its consumption in some countries and its medical use as well as low toxic potential, cannabis remains the most widely used drug around the world and the rate of usage is only increasing.

Nevertheless, there are several case reports of vascular complications following cannabis use even in young people without cardiovascular risk factors. We report the case of a cannabis smoker presenting to the emergency room for an ischemic stroke associated with an acute coronary syndrome related to a spontaneous simultaneous double dissection of the carotid artery and the left anterior descending artery, with a favourable outcome under medical treatment. This case shows the seriousness of complications due to the cannabis consumption, hence the need to limit or even prohibit its consumption.

## Introduction

Cannabis, known as marijuana, is the most widely used illicit drug in the world. Its consumption is steadily increasing due to its legalization in several countries and its recreational and medical use ^1^.

Although the mechanisms are not yet well established, the devastating effect of cannabis abuse on the cardiovascular system, even in the absence of other cardiovascular risk factors, is demonstrated.

Diverse cases of cannabis-related acute coronary syndrome (ACS), ischemic strokes or vascular attacks associated with cannabis use have been reported ^2^.

Herein, we present the first case of cannabis-induced spontaneous simultaneous double coronary and carotid dissection.

## Case report

 A 32-year-old Caucasian student male was admitted to our intensive care unit (ICU) for right total hemiplegia and aphasia evolving for 4 hours associated with chest discomfort.

His past medical history revealed no cardiovascular risk factors or symptoms. He was occasional cannabis smoker and reported daily consumption in the last 5 days.

 An initial exam showed stable hemodynamic parameters, the patient was conscious and executed orders successfully with his left extremities, but he had an incomprehensive verbal response. An electrocardiogram showed ST-segment elevation in the anterior leads, compatible with an ST-elevation myocardial infarction (STEMI).

**Figure 1.**
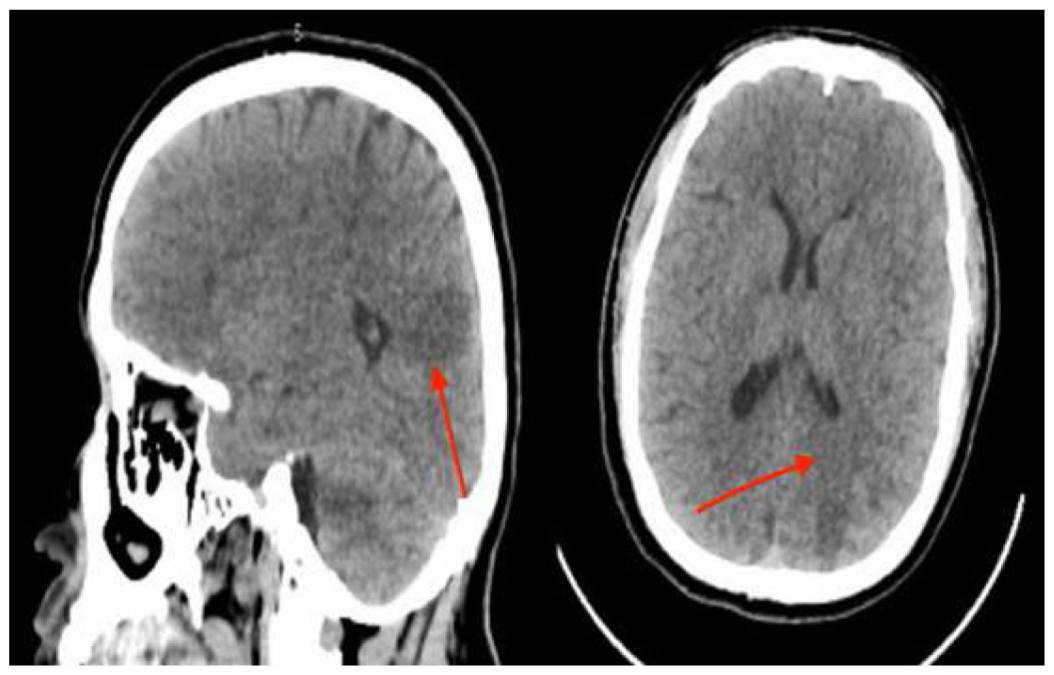
contrast-enhanced computed tomography (CT) scan showed embolic cerebral infraction consisting in multiple supratentorial regions of hypodensity in a vascular distribution occurring in the white matter-gray-matter.

**Figure 2.**
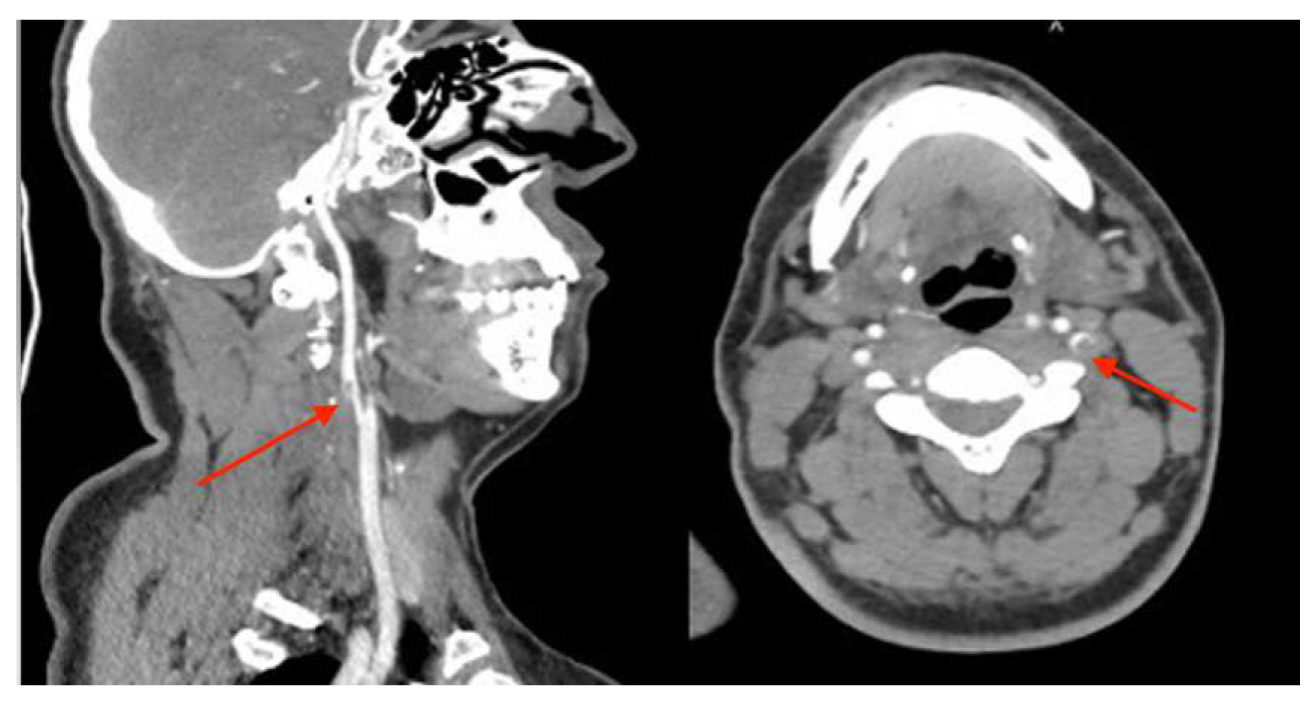
contrast-enhanced computed tomography (CT) scan showed in the cervicothoracic section a thrombosed dissection of left internal carotid.

Emergent contrast-enhanced computed tomography (CT) scan showed spontaneous hyper-density regarding the left frontal cortex, a sub-cortical left frontal, and multiple supratentorial regions of hypodensity in a vascular distribution occurred in the white matter- grey-matter. ( Figure 1) In the cervicothoracic section, it showed thrombosed dissection of the left internal carotid artery, extending over 21 mm in height. ( Figure 2)

The evolution was marked by spontaneous regression within 30 minutes of ST segment elevation and appearance of anterior negative T waves.

Echocardiography showed limited left ventricular anterior and apical wall motion abnormalities with conserved systolic ejection fraction (LVEF: 55%). The laboratory results were normal except for elevated cardiac enzymes.

**Figure 3.**
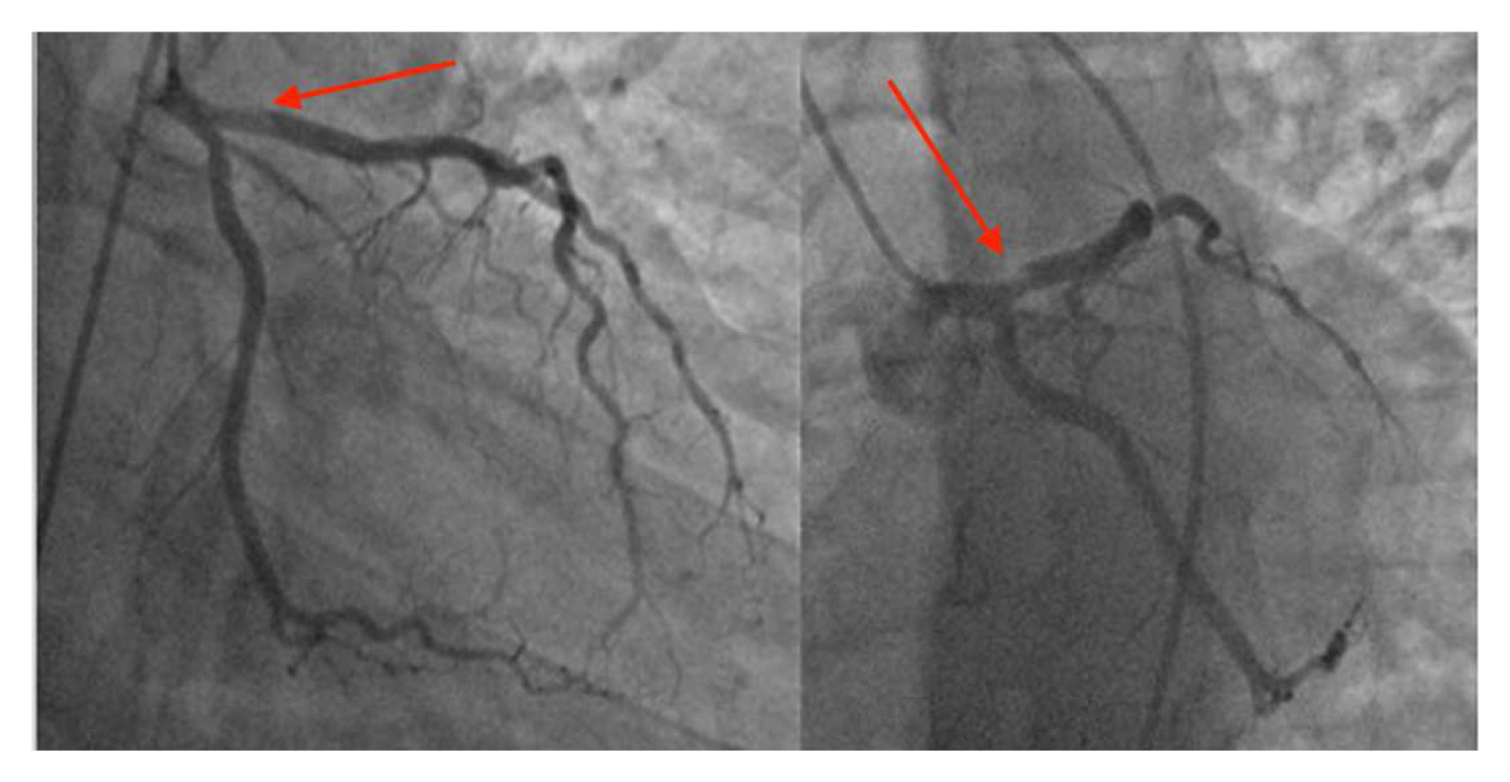
Coronary angiography showed an acute thrombotic type 1 dissection of the proximal left descending artery with TIMI III blood flow.

 Early cardiac catheterization showed an acute thrombotic dissection of the proximal left descending artery with TIMI III blood flow. ( Figure 3) The circumflex artery and the right coronary artery were normal. We decided to respect the lesion, and to put him under double antiplatelet therapy (clopidogrel 75 mg per day, aspirin 160 mg per day), unfractionated heparin, nitrates, and bisoprolol.

A second cerebral CT scan followed 48 hours later and showed favourable evolution of the cerebral lesions, so we decided to continue conservative treatment with close follow up. In-hospital outcome was favourable, with regression of aphasia and hemiplegia starting from the fifth day of the hospitalization. He was discharged after 15 days on clopidogrel 75 mg per day, aspirin 160 mg per day, atorvastatin 40 mg per day, and bisoprolol 2.5 mg per day.

At 3-month clinical control check-up, he retained right lower limb motor sequelae. Cardiac control showed the absence of symptoms, with a good electrocardiographic and echocardiographic evolution (LVEF: 60–65%).

## Discussion

Marijuana consumption has been considered benign for a long time, but multiple cardiovascular effects have been described ^3^.

At low or moderate doses, smoked marijuana increases sympathetic activity and reduces parasympathetic activity resulting in tachycardia, hypertension and may induce atrial fibrillation ^4^. conversely, high doses cause bradycardia and hypotension ^5^.

 In experimental conditions, cannabis causes arteriolar vasodilation which probably explains its low toxic potential, however, this is not always the rule. Contrasting effects of cannabinoids have been shown, responsible for vasoconstriction with acute coronary syndrome, stroke, or peripherical arteriopathy as complications ^6^.

Marijuana abuse can cause myocardial ischemia by various mechanisms including rupture of high-risk pre-existing plaques ^7^, coronary vasospasm, and coronary embolism ^8^.

Hemodynamic and oxidative stress weakens arterial walls and promotes plaque rupture thus allowing platelet activation, thrombus formation, and infarction ^9, 10^

Arterial fragility in our case as a consequence of oxidative stress, is the most logical mechanism explaining spontaneous double dissection.

 A recent systematic review of all cases of cannabis-induced myocardial infarction, showed male predominance (95,2%). Angiographic findings reveal involved occluded coronary arteries in 63,2% whose involvement concerned the left anterior descending artery in 42.1% of cases. Conservative management with medical observation was sufficient in 46.7% of patients ^2^.

Cerebrovascular ischemic lesions are common in marijuana abuse where reversible cerebrovascular spasm is most often the cause ^11^.

Spontaneous artery dissection is a rare cause of ACS or stoke. It is generally associated with particular clinical situations: the use of contraceptives, pregnancy, Marfan syndrome, connective tissue disorders, trauma, and cocaine abuse ^12^.

Several cases of cannabis-induced dissection have been reported in the literature, affecting coronary and cerebral arteries but also the aorta and renal arteries ^13, 14^.

No previous case of spontaneous simultaneous coronary and carotid dissection related to cannabis use has been reported.

There is no proven therapeutic strategy for cannabis-related dissection as the literature is limited to case studies. Double antiplatelet therapy without vascular intervention can be attempted in hemodynamically stable patients ^2^.

## Conclusion

This case highlights cannabis-related coronary and cerebral complications in in early adulthood.

An increase in such cases is to be expected in the face of legalization of cannabis consumption and medical use which is spreading in several countries. If the increase in global consumption becomes unavoidable, the search for predictive factors of complications due to cannabis consumption appears necessary to avoid these serious consequences.

## Patient consent

Written informed consent for publication of their clinical details and/or images was obtained from the patient.

## Data availability statement

All data underlying the results are available as part of the article and no additional source data are required.
