# Supplementary material for: Case Report: Spontaneous simultaneous coronary and carotid dissection in a young cannabis user
Source: F1000Res. 2021 Jun 11;10:387. Originally published 2021 May 14. [Version 2] doi: 10.12688/f1000research.52606.2 (PMC8204189; doi:10.12688/f1000research.52606.2)
Supplement: Peer review history

# Peer review: Reviewer response for version 2

**DOI:** 10.5256/f1000research.57669.r87200

**Published:** 2021-06-14

**Authors:** Amr Ashry

**Affiliations:** 1 Department of Paediatric Cardiac Surgery, Alder Hey Children Hospital, Liverpool, UK; 2 Department of Cardiothoracic Surgery, Faculty of Medicine, Assiut University, Assiut, Egypt

The authors have written an interesting case report about a young male patient who is a cannabis user who developed spontaneous simultaneous double dissection of the carotid artery and the left anterior descending coronary artery. I congratulate the authors for their effort in writing such a rare case in attractive way with clear images of the investigations that were done.The authors also mentioned the success of the conservative management with dual anti platelets therapy. The discussion part is well written in an organised way that will definitely attract the readers. In general, I found the case report interesting and I approve it for indexing.

Are enough details provided of any physical examination and diagnostic tests, treatment given and outcomes?

Yes

Is the case presented with sufficient detail to be useful for other practitioners?

Yes

Is sufficient discussion included of the importance of the findings and their relevance to future understanding of disease processes, diagnosis or treatment?

Yes

Is the background of the case’s history and progression described in sufficient detail?

Yes

Reviewer Expertise:

Cardiac surgery, cardiology and thoracic surgery.

I confirm that I have read this submission and believe that I have an appropriate level of expertise to confirm that it is of an acceptable scientific standard.

# Peer review: Reviewer response for version 2

**DOI:** 10.5256/f1000research.57669.r87364

**Published:** 2021-06-14

**Authors:** Rania Hammami

**Affiliations:** 1 Cardiology Department, Hedi Chaker University Hospital, University of Medicine of Sfax, Sfax, Tunisia

I would like to thank the authors for these corrections. The paper in this status could be accepted.

Are enough details provided of any physical examination and diagnostic tests, treatment given and outcomes?

Yes

Is the case presented with sufficient detail to be useful for other practitioners?

Yes

Is sufficient discussion included of the importance of the findings and their relevance to future understanding of disease processes, diagnosis or treatment?

Yes

Is the background of the case’s history and progression described in sufficient detail?

Yes

Reviewer Expertise:

interventionnal cardiologist

I confirm that I have read this submission and believe that I have an appropriate level of expertise to confirm that it is of an acceptable scientific standard.

# Peer review: Reviewer response for version 1

**DOI:** 10.5256/f1000research.55907.r85461

**Published:** 2021-06-01

**Authors:** Rania Hammami

**Affiliations:** 1 Cardiology Department, Hedi Chaker University Hospital, University of Medicine of Sfax, Sfax, Tunisia

I would like first to thank the authors for this interesting case of coronary and carotid Spontaneous dissection in a young patient after cannabis consumption! After reviewing the literature, this clinical case is the first to show a spontaneous and simultaneous double dissection (of coronary and carotid arteries).

 The clinical case highlights the seriousness of cannabis-related cardiovascular complications.

 Cannabis is the most widely used illicit drug in the world, its consumption is legalized in some countries.

 Cannabis consumption, long considered to have a low cardiovascular risk, is sometimes responsible for serious complications in young subjects with significant sequelae and functional limitation, thus encouraging to limit or even prohibit its marketing.

 The second strong point is to show the interest of a medical treatment comprising a double antiplatelet therapy in the treatment of these cases.

 The discussion is rich. The authors described the cardiovascular complications associated with cannabis as described in the literature and their management with recent references on this subject.

 Figures are clear with visible dissection images in the carotid artery and the proximal left descending artery. I suggest adding an arrow to the pictures. Did you perform a cerebral or coronary imaging control in this patient, if it was the case, please add the pictures?

Are enough details provided of any physical examination and diagnostic tests, treatment given and outcomes?

Yes

Is the case presented with sufficient detail to be useful for other practitioners?

Yes

Is sufficient discussion included of the importance of the findings and their relevance to future understanding of disease processes, diagnosis or treatment?

Yes

Is the background of the case’s history and progression described in sufficient detail?

Yes

Reviewer Expertise:

interventionnal cardiologist

I confirm that I have read this submission and believe that I have an appropriate level of expertise to confirm that it is of an acceptable scientific standard.
